# Cardiosphere-Derived Cells Require Endoglin for Paracrine-Mediated Angiogenesis

**DOI:** 10.1016/j.stemcr.2017.04.015

**Published:** 2017-05-09

**Authors:** Rachael E. Redgrave, Simon Tual-Chalot, Benjamin J. Davison, Esha Singh, Darroch Hall, Muhammad M. Amirrasouli, Derek Gilchrist, Alexander Medvinsky, Helen M. Arthur

**Affiliations:** 1 Institute of Genetic Medicine, Centre for Life, Newcastle University, Newcastle NE1 3BZ, UK; 2 Institute for Stem Cell Research, MRC Centre for Regenerative Medicine, Scottish Centre for Regenerative Medicine, University of Edinburgh, 5 Little France Drive, Edinburgh EH16 4UU, UK

## Abstract

Clinical trials of stem cell therapy to treat ischemic heart disease primarily use heterogeneous stem cell populations. Small benefits occur via paracrine mechanisms that include stimulating angiogenesis, and increased understanding of these mechanisms would help to improve patient outcomes. Cardiosphere-derived-cells (CDCs) are an example of these heterogeneous stem cell populations, cultured from cardiac tissue. CDCs express endoglin, a co-receptor that binds specific transforming growth factor β (TGFβ) family ligands, including bone morphogenetic protein 9 (BMP9). In endothelial cells endoglin regulates angiogenic responses, and we therefore hypothesized that endoglin is required to promote the paracrine pro-angiogenic properties of CDCs. Cre/*LoxP* technology was used to genetically manipulate endoglin expression in CDCs, and we found that the pro-angiogenic properties of the CDC secretome are endoglin dependent both in vitro and in vivo. Importantly, BMP9 pre-treatment of endoglin-depleted CDCs restores their pro-angiogenic paracrine properties. As BMP9 signaling is normally required to maintain endoglin expression, we propose that media containing BMP9 could be critical for therapeutic CDC preparation.

## Highlights

• It is essential to understand how stem cell populations generate paracrine benefit
• Endoglin is necessary for the pro-angiogenic properties of the CDC secretome
• Pro-angiogenic defects of endoglin-depleted CDCs can be rescued by BMP9

## Teaser

In this article, Arthur and colleagues show how expression of the TGFβ co-receptor endoglin is essential for the paracrine pro-angiogenic properties of cardiosphere-derived cells, a heterogeneous stem cell population derived from the heart.

## Introduction

Stem cells have both differentiation capacities and paracrine effects that can be harnessed to promote tissue regeneration. Over the last decade, a range of heterogeneous stem cell populations including mesenchymal stem cells (MSCs) and cardiac stem cells (CSCs) have been used in clinical trials of autologous cell-based therapy to treat ischemic heart disease (Madonna et al., 2016). Although these cells show low engraftment and poor differentiation abilities in vivo, there is a consensus that they are safe and have the capacity to promote small improvements in heart function primarily via paracrine mechanisms. However, in all cases these mechanisms are poorly characterized. Moving forward, if the nature of these paracrine mechanisms can be better understood, the prospects of improving their efficacy may be considerably increased. In particular, improving their pro-angiogenic paracrine properties will be critical for treating ischemic disease.

Cardiospheres (CSps) are one of these heterogeneous stem cell populations, and form spontaneously from cultured cardiac biopsies (Davis et al., 2009, Messina et al., 2004). CSps comprise self-assembling stem cell clusters with the potential to differentiate to myocardial cell subtypes (Li et al., 2011, Smith et al., 2007). They have been shown to promote cardiac repair following myocardial infarction (MI) via paracrine mechanisms such as promoting angiogenesis and cardiomyocyte proliferation in the recipient tissue (Chimenti et al., 2010, Li et al., 2012). CSp cellular clusters are relatively large (>100 μm in diameter) and intravascular delivery carries an inherent risk of precipitating microthrombotic events. Therefore, much of the work in this area has focused on cardiosphere-derived cells (CDCs), which are suspensions of single cells prepared from cultured CSps.

Both CSps and CDCs express endoglin (also known as CD105), considered a characteristic cell-surface marker of these cells (Smith et al., 2007). Endoglin is a membrane co-receptor for specific members of the transforming growth factor β (TGFβ) family of cytokines that regulate many aspects of cell function. In endothelial cells, endoglin promotes angiogenesis by regulating the balance of TGFβ signaling through ALK5 and ALK1 receptors, essentially by enhancing signaling through ALK1 and reducing signaling through ALK5 (Lebrin et al., 2004). Mice without endoglin die in embryogenesis from angiogenic defects while mice that are heterozygous for endoglin mutations (*Eng*^*+*/−^) show poor reperfusion in mouse models of hindlimb ischemic injury (Jerkic et al., 2006, Seghers et al., 2012). Furthermore, following MI, endogenous levels of vascularity within the infarct border zone is much lower in *Eng*^*+*/−^ mice compared with wild-type controls (van Laake et al., 2006). In addition to angiogenesis, endoglin is also required for vessel integrity. Patients carrying deleterious mutations in endoglin develop hereditary hemorrhagic telangiectasia (McAllister et al., 1994), a disease typified by fragile bleeding vessels and arteriovenous malformations (AVMs) (Shovlin, 2010). Consistent with a key role in endothelial cells, endothelial-specific loss of endoglin in early postnatal life leads to angiogenic defects and AVMs (Arthur et al., 2000, Mahmoud et al., 2010).

Based on its role in endothelial cells, we hypothesized that endoglin also has a pro-angiogenic role in CDCs, contributing to their paracrine pro-angiogenic effects. To address this question, we depleted endoglin from CDCs using Cre-LoxP genetics in a mouse model, and tested the corresponding effect on downstream angiogenesis responses in a wide range of in vitro and in vivo angiogenesis assays as well as a mouse model of MI.

## Results

### Endoglin Is Required for CDC-Mediated Pro-angiogenic Paracrine Effects

**Figure 1:**
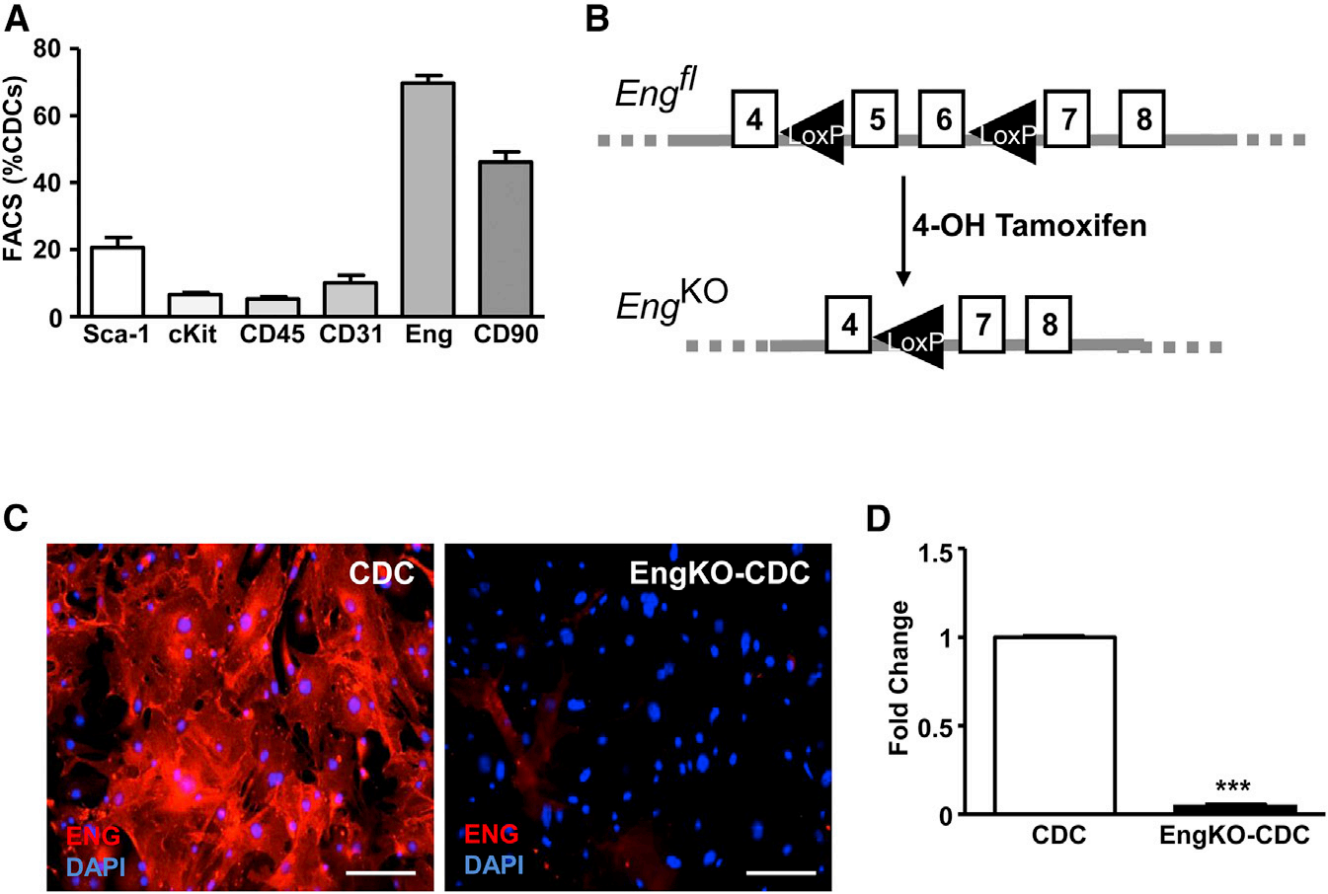
Endoglin Is Expressed in CDCs and Can Be Efficiently Depleted Using Cre-LoxP Recombination (A) Flow-cytometric analyses of murine CDCs at passage 2. Summary of the percentage of CDCs expressing mesenchymal (CD90, endoglin), hematopoietic (CD45), and stem cell (cKit, Sca-1) markers from three independent experiments and plotted as mean percentage ± SEM. (B) Summary of the floxed endoglin allele (Eng^fl^) and Cre recombination driven by the R26-Cre^ERT2^ allele in the presence of 4-hydroxytamoxifen to generate a null endoglin knockout (*Eng*^KO^) allele. (C) Immunostained CDCs showing that transient 48-hr treatment with 4-hydroxytamoxifen leads to efficient endoglin protein depletion. Scale bar, 50 μm. (D) qPCR analysis showing loss of endoglin transcripts in *Eng*^KO^ CDCs. ^∗∗∗^p < 0.001.

CDCs were characterized using fluorescence-activated cell sorting (FACS) and immunocytostaining (Figure 1). The majority of CDCs at passage 2 (P2) express endoglin, although the proportion of endoglin-positive CDCs reduces at later passages (not shown). A significant proportion (56%) of P2 CDCs also express CD90, while 4% of CDCs express the stem cell marker Kit and 10% express *Ly6a* (also known as *Sca-1*). Very few CDCs (∼2%) are CD45^+^, confirming that they are not derived from hematopoietic cells. Mice carrying the *CAG-eGFP* transgene were used to generate GFP-expressing CDCs that could be tracked over time (Figure S1). To determine the role of endoglin, we prepared CDCs from mice in which Endoglin can be depleted by Cre/*LoxP* technology. CDCs from *CAG-eGFP*;*Eng*^*fl*/*fl*^;*Rosa26-Cre*^*ERT2*^ mice were divided into two matched populations for each experiment. One CDC population was used as a wild-type control and the corresponding CDCs were transiently treated with 4-hydroxytamoxifen (4-OHT) to generate *Eng*^KO^ CDCs (Figure 1B). Endoglin loss was confirmed at the transcript and protein level by qPCR and immunocytostaining, respectively (Figures 1C and 1D). The paracrine pro-angiogenic effects of the secretome of CDCs with and without endoglin were compared using conditioned medium (CM) that had been harvested from P2 CDCs at equivalent cell densities, and checked for equal protein concentration prior to use (Figure S2A).

**Figure 2:**
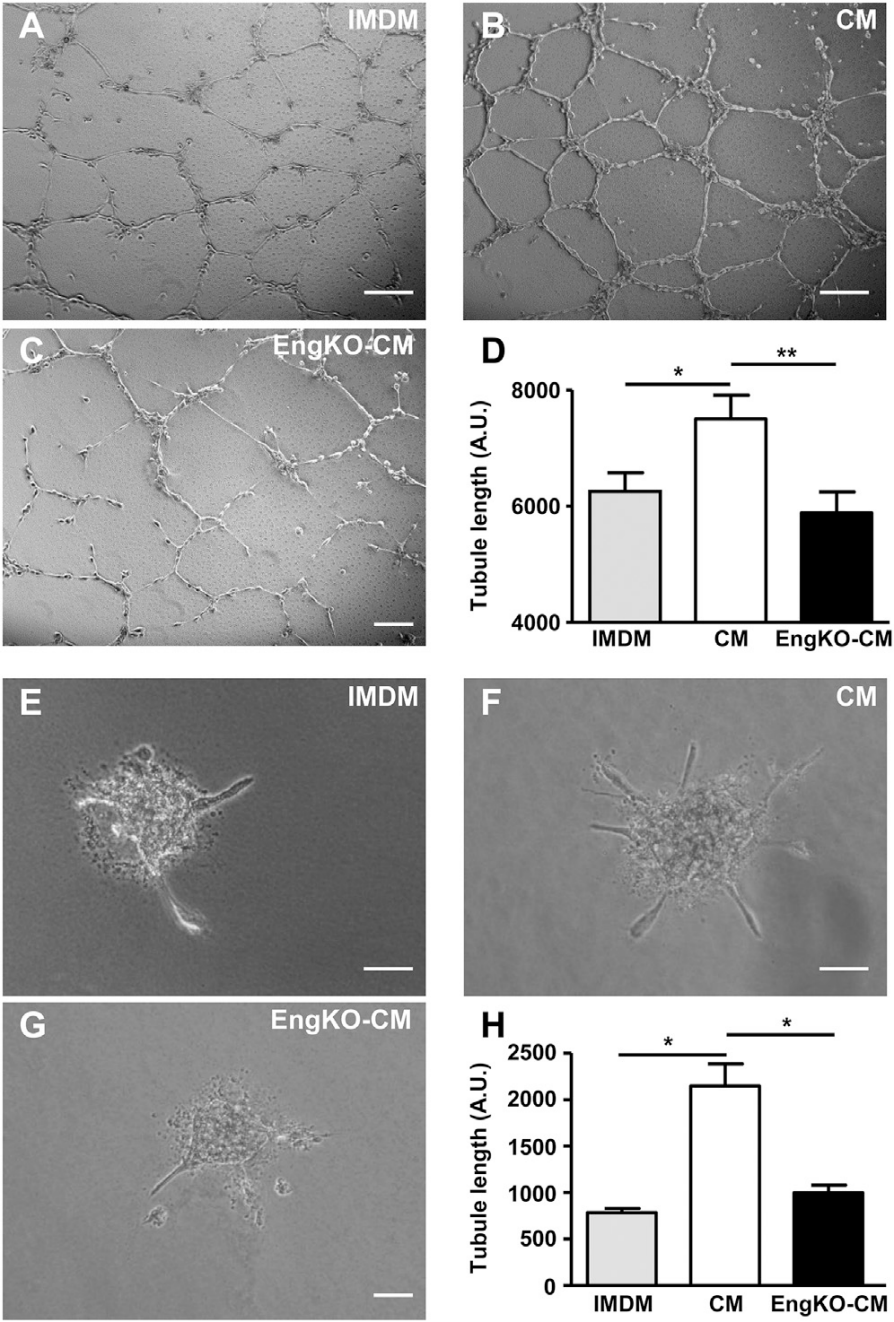
Endoglin Expression Is Required for Paracrine CDC-Mediated Pro-angiogenic Effects In Vitro (A–D) Representative phase-contrast micrographs showing the relative pro-angiogenic effects of basal medium (IMDM, A), CDC-CM (B), and *Eng*^KO^ CDC-CM (C) on HMEC tubule formation in a 2D Matrigel assay. Quantitation of tubule length (D) shows that the pro-angiogenic effect of CDC-CM is lost in the absence of endoglin. Data are plotted as mean ± SEM from eight independent experiments; ^∗^p < 0.05, ^∗∗^p < 0.01. Scale bar, 200 μm. (E–H) Representative phase-contrast micrographs showing angiogenic sprouting of HUVEC spheroids in the presence of basal medium (E), control CM (F), or *Eng*^KO^ CM (G). Angiogenesis promoted by control CM is lost in *Eng*^KO^ CM (H). Data from three independent experiments with 20 spheroids per group are shown as mean ± SEM; ^∗^p < 0.05. Scale bar, 100 μm.

**Figure 3:**
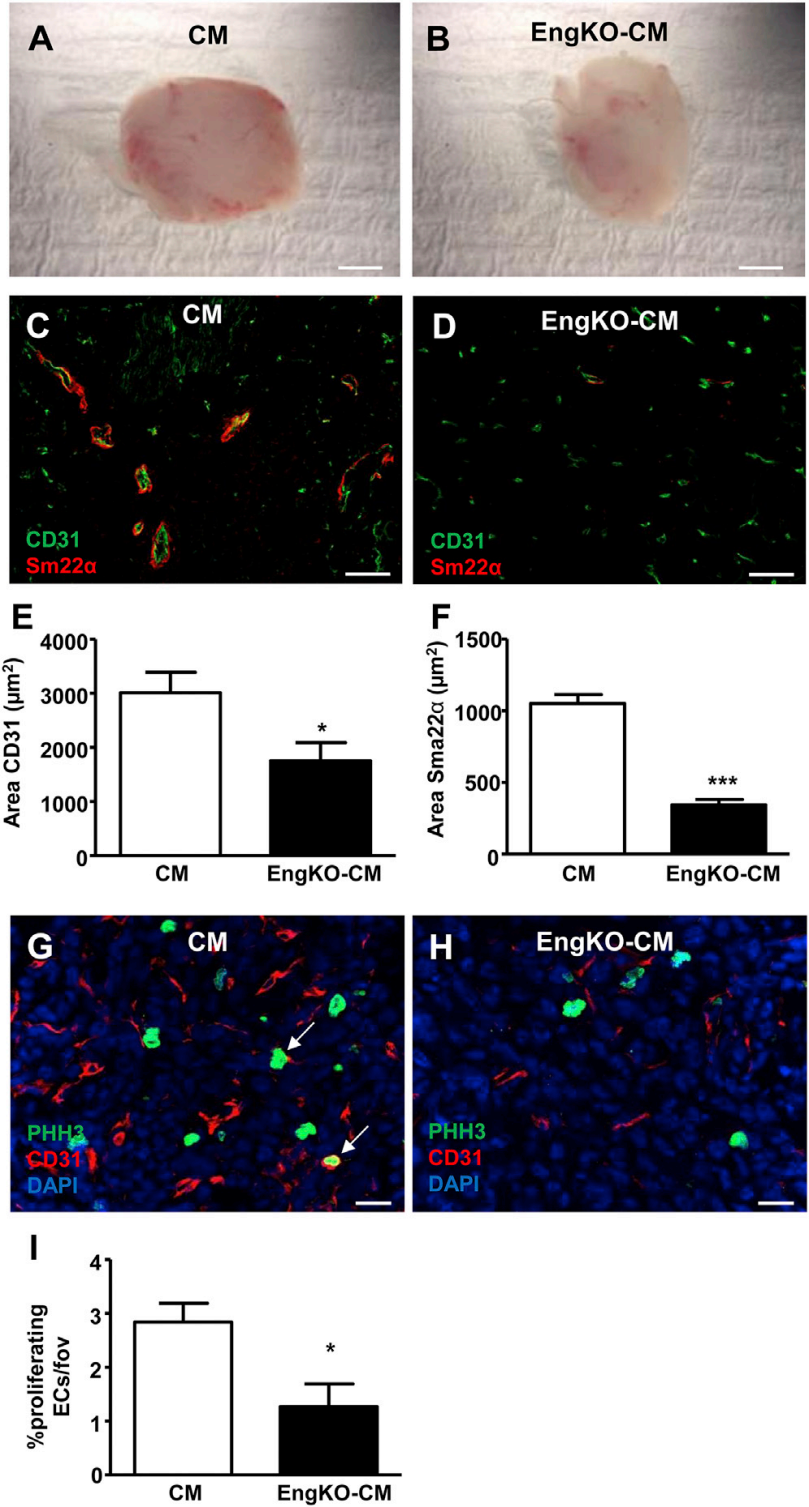
Endoglin Expression Is Required for Paracrine CDC-Mediated Pro-angiogenic Effects in 3D Matrigel Plugs In Vivo (A and B) Whole-mount view of freshly dissected Matrigel plugs at 14 days after seeding with MLECs and either CM (A) or *Eng*^KO^ CM (B) shows decreased vascularity of *Eng*^KO^ CM plugs. Scale bar, 3 mm. (C–F) Cryosections of Matrigel plug immunostained with anti-CD31to detect endothelial cells (green) and anti-SM22α to detect vascular smooth muscle cells (red) shows decreased vascularity of Matrigel plugs containing *Eng*^KO^ CM (D) compared with CM (C). Scale bar, 100 μm. Quantification of CD31 (E) and SM22α (F) staining in six Matrigel plugs per group using ImageJ. ^∗^p < 0.05, ^∗∗∗^p < 0.001. (G–I) Matrigel plug sections immunostained with anti-phospho-histone-3 Ser10 (pH3) to detect proliferating cells (green) and anti-CD31 to detect endothelial cells (red) shows proliferating endothelial cells (arrows, G). There are fewer proliferating endothelial cells in plugs containing *Eng*^KO^ CM (H) compared with CM (G). Nuclei of all cells are counterstained with DAPI (blue) Scale bar, 20 μm. Data are quantified as percentage proliferating endothelial cells (I); n = 6 Matrigel plugs per group; ^∗^p < 0.05.

The pro-angiogenic effects of the secretomes from *Eng*^KO^ and control CDCs were compared in a range of angiogenesis assays using both in vitro and in vivo conditions. First, using the human microvascular endothelial cell 1 (HMEC-1) cell line in a 2D Matrigel angiogenesis assay, we observed that control CDC-CM significantly enhanced endothelial tubule formation compared with basal medium. However, this pro-angiogenic effect of CDC-CM was lost when CM was prepared from *Eng*^KO^ CDCs (Figures 2A–2D). Similarly, using human umbilical vein endothelial cells (HUVECs) in a spheroid angiogenesis assay, the pro-angiogenic effect of CDC-CM was absent following endoglin depletion from CDCs (Figures 2E–2H). Importantly, using CDCs cultured from C57BL/6 mice, we confirmed that 4-OHT treatment alone has no effect on the pro-angiogenic properties of CDC-CM in this assay (data not shown). In addition, a subdermal Matrigel plug assay was used to test whether the pro-angiogenic paracrine effects of CDCs were endoglin-dependent in vivo. In this assay, endothelial cells form a functional vascular network in a subdermal Matrigel plug over a 2-week time course. Matrigel plugs seeded with CM from *Eng*^KO^ CDCs showed significantly reduced vessel formation compared with CM from control CDCs (Figures 3A–3F). In addition, endothelial cell proliferation was reduced in plugs seeded with CM from *Eng*^KO^ CDCs compared with control CM (Figures 3G–3I), confirming that endoglin was required for the pro-proliferative paracrine effects of CDCs in vivo. Furthermore, we observed reduced numbers of mature muscularized vessels in Matrigel plugs containing *Eng*^KO^ CDC-CM compared with control CDC-CM, indicating that the pro-angiogenic effect of CDC-CM was of lasting duration that led to mature muscularized vessels, and that this was also endoglin dependent (Figure 3F).

### Endoglin Is Required for CDC-Mediated Pro-angiogenic Effects after Myocardial Infarction

**Figure 4:**
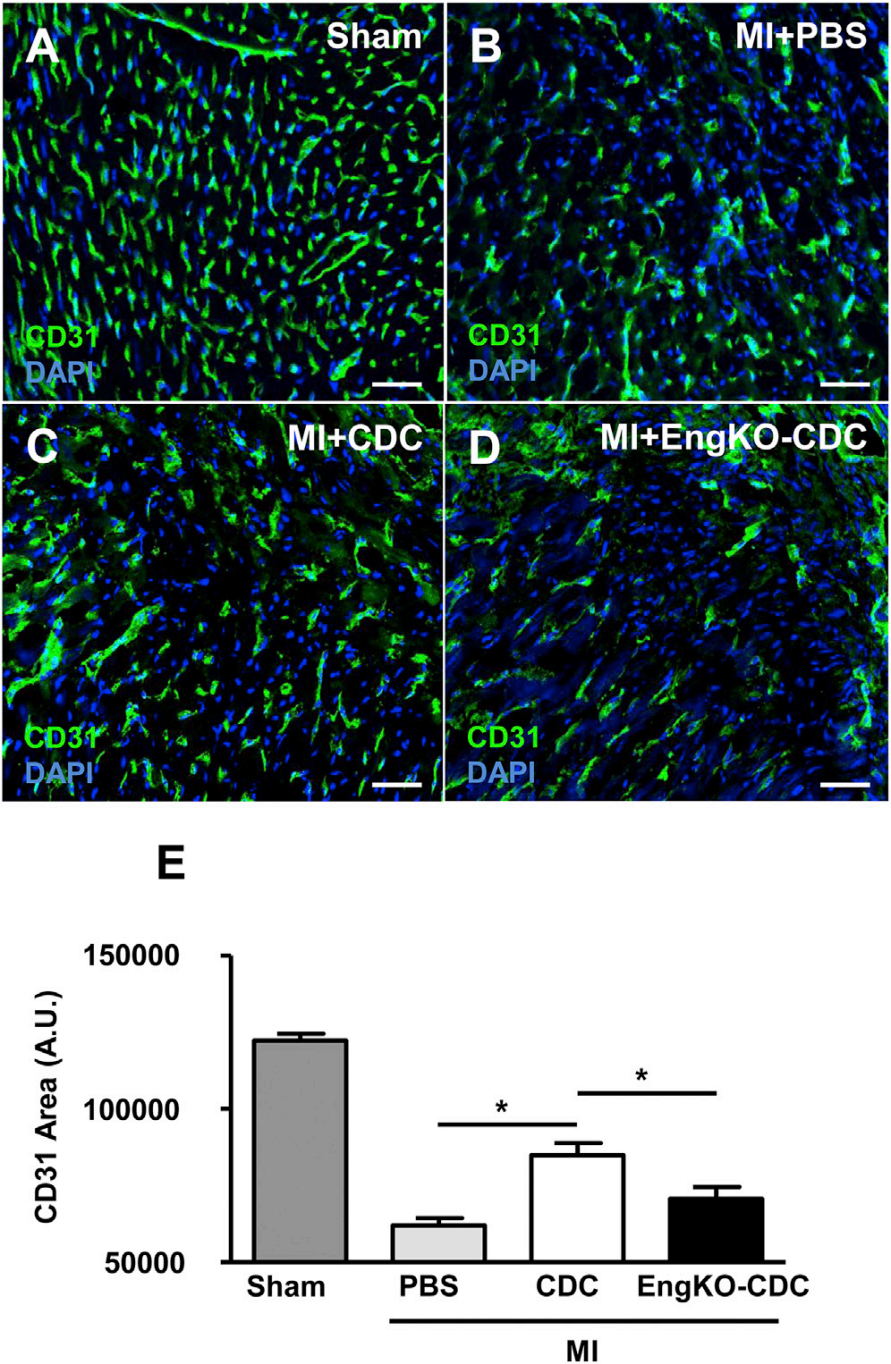
Endoglin Is Required for CDC-Mediated Pro-angiogenic Effects in the Infarct Border Zone of the Heart after Myocardial Infarction (A–E) Vessel density in the border zone of MI hearts was analyzed using CD31 immunostaining at 4 weeks post injury in mice that had sham operation (A), MI with PBS injection (B), MI with CDC injection (C), and MI with *Eng*^KO^ CDC injection (D). Scale bar, 50 μm. Area of CD31 immunofluorescence was quantified in 20 fields of view per heart and analyzed with ImageJ software. n = 5 sham, n = 8 MI + PBS, n = 8 MI + CDC, and n = 7 MI + *Eng*^KO^ CDC; ^∗^p < 0.05.

To determine whether the pro-angiogenic effect of CDCs following MI was also endoglin dependent, we subjected male C57BL/6 mice to a surgical MI and injected CDCs into the infarct border zone. All CDCs were prepared from *CAG-eGFP*;*Eng*^*fl*/*fl*^;*Rosa26-Cre*^*ERT2*^ permitting generation of matched control and *Eng*^KO^ CDCs, and intracardiac injections were performed by a surgeon blinded to CDC genotype. Hearts were harvested after 4 weeks to evaluate stable vascularity in the infarct border zone. Delivery of control CDCs significantly increased the vascularity of the infarct border zone, consistent with the reported paracrine pro-angiogenic effect of CDCs in vivo (Chimenti et al., 2010). However, endoglin-depleted CDCs had little benefit, generating significantly reduced vessel density in the infarct border zone compared with hearts injected with control CDCs (Figures 4A–4E). These findings were consistent with our earlier data showing that the pro-angiogenic effect of CDCs was endoglin dependent. In addition, any direct contribution of CDCs from *CAG-eGFP* donor mice to the vessels of the wild-type recipient hearts was investigated using anti-GFP immunostaining. Direct contribution of GFP-labeled CDCs to these vessels was rarely observed, confirming the pro-angiogenic effect of CDCs were a result of paracrine mechanisms (Figures S2B and S2C).

Heart function was measured using cardiac magnetic resonance imaging (MRI) at 1 week and again at 4 weeks following MI, but the pro-angiogenic effect of CDCs was insufficient to promote significant rescue of cardiac function, in line with the large infarct size used in this study (Figure S3). Left ventricular mass, ejection fraction, and end-diastolic and systolic volumes at 1 week and at 4 weeks following MI were similar in both CDC-treated and CDC-untreated mice, irrespective of the presence of endoglin (Figure S3).

As endoglin acts as a co-receptor for bone morphogenetic protein 9 (BMP9), BMP10, TGFβ1, and TGFβ3 ligands (Castonguay et al., 2011, Cheifetz et al., 1992, Scharpfenecker et al., 2007), we used a separate group of wild-type C57BL/6 mice (without CDCs) to examine the relative levels of these ligands in infarcted myocardium, as well as in serum, during the first week after MI. This time period was chosen as it corresponds to the period immediately after CDC injection, when the ligands would be available to interact with endoglin-expressing CDCs, before CDCs are cleared from the recipient heart tissue. Neither BMP9 nor BMP10 were detected by qPCR in the left ventricular tissue either before or after MI (data not shown). In contrast, qPCR revealed dynamic changes in expression of TGFβ1 and TGFβ3 in the left ventricular tissue following MI, reaching a peak at day 5 for TGFβ1 and at day 7 for TGFβ3 (Figure S4), similar to previously published data (Christia et al., 2013, Deten et al., 2001). In addition, TGFβ1, BMP9, and BMP10 ligands are present in serum throughout this period, both in MI and sham groups. Circulating TGFβ1 protein levels remain unchanged, but BMP9 concentrations significantly decrease and BMP10 concentrations significantly increase in the days following surgery (Figure S4). TGFβ3 protein was not detectable in serum from any of the mice (data not shown). The availability of TGFβ1, BMP9, and BMP10 protein in serum and increased TGFβ1 and TGFβ3 expression in infarcted myocardium means that the presence of endoglin in CDCs is biologically relevant during the cardiac healing process.

### Endoglin Is Required for CDC-CM to Promote Endothelial Cell Migration but Does Not Affect VEGF Signaling

**Figure 5:**
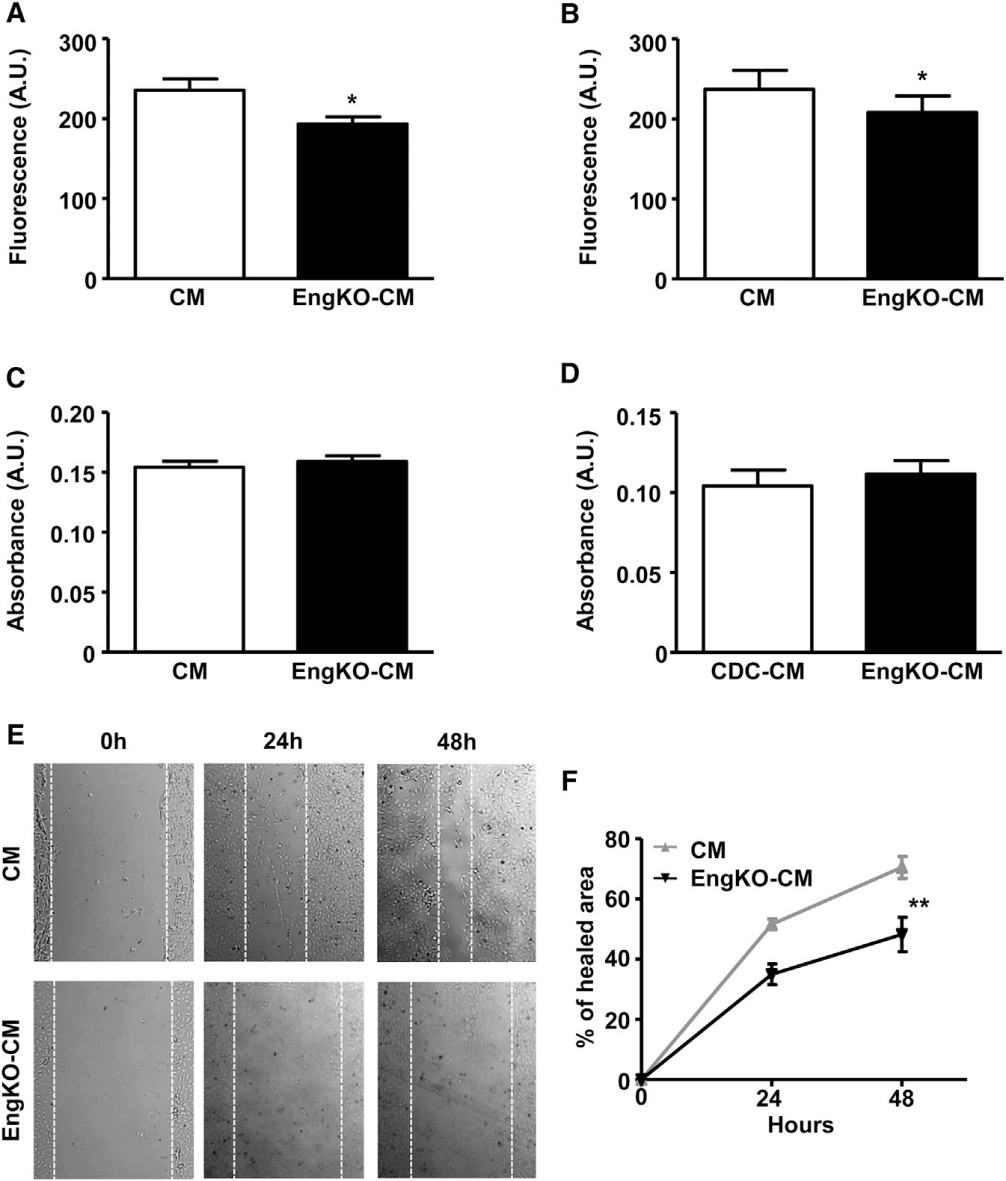
Endoglin Is Required to Maximize Pro-proliferative and Pro-migratory Endothelial Cell Responses (A and B) Proliferation of HUVECs measured using a fluorometric assay (CyQUANT) at 24 hr (A) and 48 hr (B) is reduced in the presence of *Eng*^KO^ CM compared with control CM. Data from nine independent experiments are represented as mean ± SEM; ^∗^p < 0.05. (C and D) Viability of HUVECs was measured using a colorimetric (MTT) assay and is similar following treatment with control CM or *Eng*^KO^ CM for 24 hr (C) and 48 hr (D). Data from seven independent experiments are represented as mean ± SEM. (E and F) HUVEC migration in the scratch-wound assay is significantly reduced in the presence of *Eng*^KO^ CM compared with control CM. Data from five independent experiments are plotted as mean ± SEM, ^∗∗^p < 0.01.

As endoglin was required for the pro-angiogenic effects of CDCs, we next sought to investigate the mechanism. Vascular endothelial growth factor A (VEGFA) is the “master” pro-angiogenic factor, and crosstalk occurs between endoglin and VEGF pathways (Liu et al., 2014). Therefore, altered VEGF signaling could potentially explain the reduced angiogenic response seen in *Eng*^KO^ CDC-CM. However, we observed no difference in VEGFA content of CM from control or *Eng*^KO^ CDCs (Figure S5). Furthermore, HUVECs treated with control CM and *Eng*^KO^ CM showed similar phospho-AKT and phospho-GSK responses (Figure S5), consistent with comparable signaling responses to VEGF. As angiogenesis results from increased proliferation and migration of endothelial cells, we next tested the effect of CDC-CM on these endothelial responses. There was a small decrease in HUVEC proliferation in the presence of *Eng*^KO^ CDC-CM compared with control CDC-CM (Figures 5A and 5B), but no change in HUVEC viability in the presence of control or *Eng*^KO^ CDC-CM (Figures 5C and 5D). However, “scratch-wound” closure was significantly slower in the presence of *Eng*^KO^ CDC-CM compared with control CDC-CM (Figures 5E and 5F), consistent with a reduced pro-migratory effect of *Eng*^KO^ CDC-CM. Taken together, this evidence points to the requirement of endoglin in CDCs to promote the pro-proliferative and pro-migratory paracrine effects of CDC-CM on endothelial cells.

### Endoglin-Dependent Expression of Pro- and Anti-angiogenic Factors in the CDC Secretome

**Figure 6:**
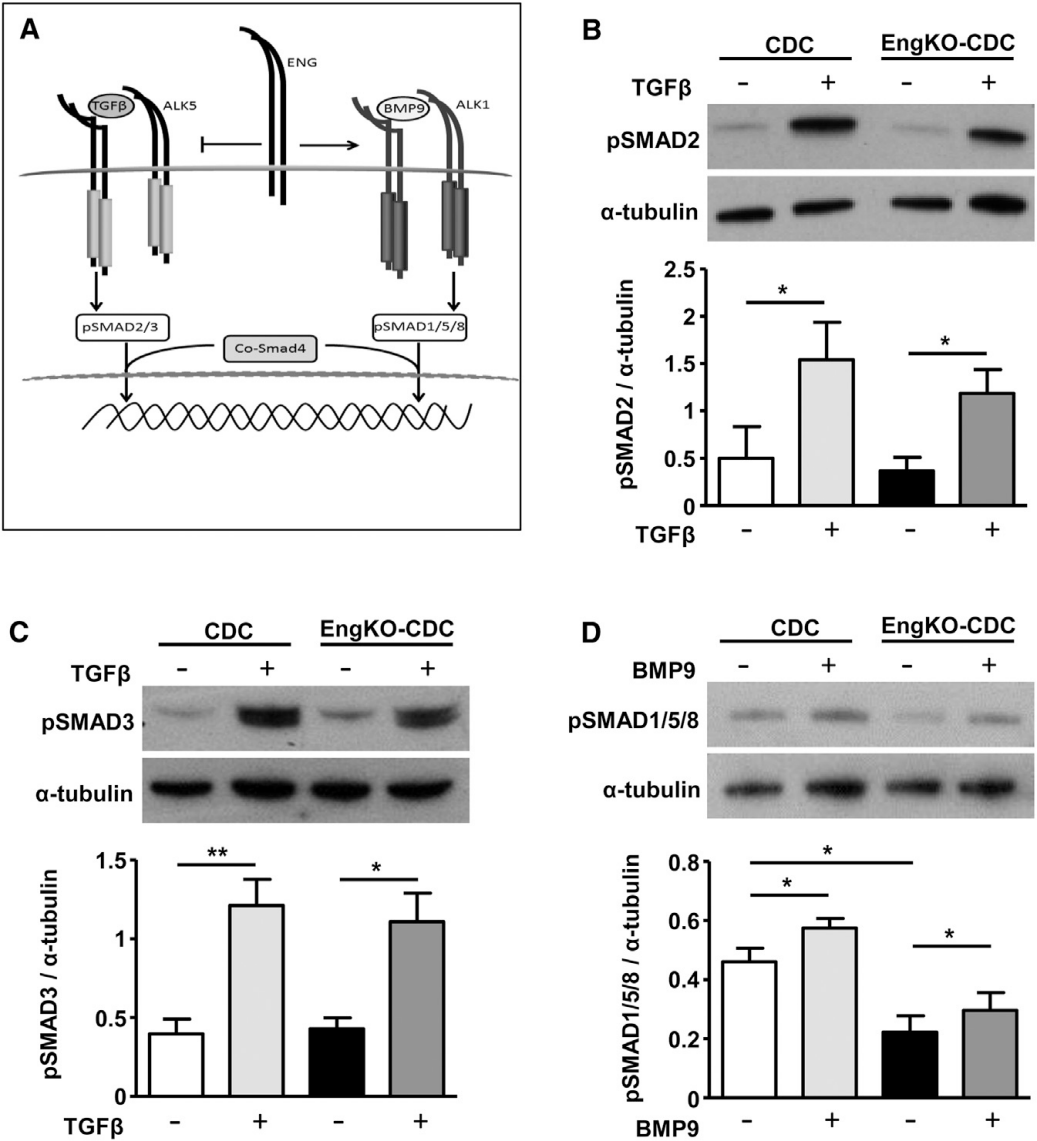
Endoglin-Deficient CDCs Show Reduced Endogenous SMAD1/5/8 Activation that Can Be Rescued by BMP9 Treatment (A) Summary of TGFβ and BMP9 signaling pathways. TGFβ/ALK5 and BMP9/ALK1 signaling are propagated through phosphorylation of SMAD2/3 and SMAD1/5/8, respectively. TGFβ generally signals via a complex of TGFBR2 and ALK5 (upper left) while BMP9 (and BMP10) signals via a complex of BMPR2 and ALK1 (upper right). Endoglin acts as a co-receptor to promote BMP9/10 signaling through the ALK1 receptor complex, although it can also promote TGFβ signaling through ALK1 to activate SMAD1/5/8 (not shown). (B and C) Representative western blots show that baseline and TGFβ1-induced SMAD2 phosphorylation (B) and SMAD3 phosphorylation (C) are similar in control and in *Eng*^KO^ CDCs. Densitometric analysis of pSMAD2 and pSMAD3 band intensity relative to α-tubulin are shown as mean ± SEM from three independent experiments. ^∗^p < 0.05; ^∗∗^p < 0.01. (D) Representative western blot shows SMAD1/5/8 phosphorylation is reduced in *Eng*^KO^ CDCs and this is partially rescued following treatment with 2 ng/mL BMP9 for 30 min. Densitometric analysis of pSMAD1/5/8 band intensity relative to α-tubulin is shown as mean ± SEM from five independent experiments; ^∗^p < 0.05.

**Figure 7:**
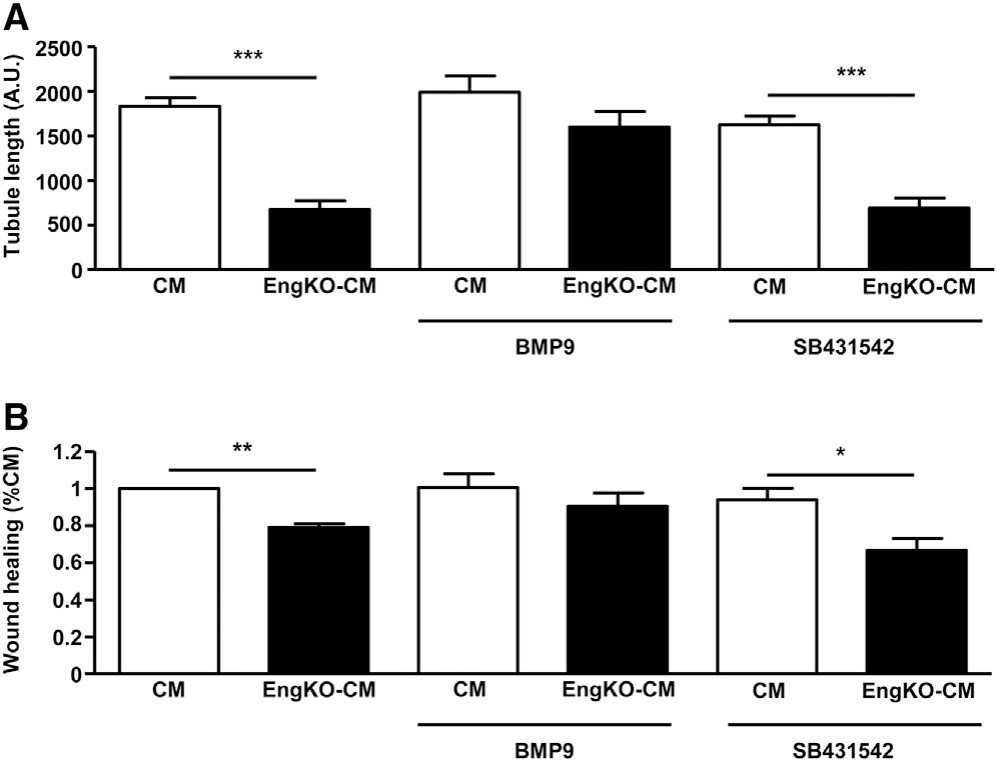
Pre-treatment of CDCs with BMP9 Rescues the Paracrine Pro-angiogenic Defects Caused by Endoglin Depletion (A) *Eng*^KO^ CM shows reduced pro-angiogenic effects compared with CM from control CDCs in the HUVEC spheroid assay. Pre-treatment of CDCs with 2 ng/mL of BMP9 for 24 hr prior to generating CM led to rescue of the *Eng*^KO^ CM pro-angiogenic defect. In contrast, pre-treatment with ALK5 inhibitor (SB431542) had no effect on the EngKO CM pro-angiogenic defect. Data are plotted as mean ± SEM from three independent experiments; ^∗∗∗^p < 0.001. (B) Healing of the HUVEC scratch-wound assay is reduced in the presence of *Eng*^KO^ CM. CM collected after pre-treatment of *Eng*^KO^ CDCs with 2 ng/mL BMP9 for 24 hr restored the pro-migratory capacity of *Eng*^KO^ CM. In contrast, pre-treatment with ALK5 inhibitor (SB431542) had no effect. Data are plotted as mean ± SEM from three independent experiments; ^∗^p < 0.05, ^∗∗^p < 0.01.

Endoglin promotes signaling through ALK1 and reduces signaling through ALK5 in endothelial cells to promote angiogenesis (Figure 6A) (Lebrin et al., 2004). Also, CDCs express the receptor profile required for activation of both ALK1 and ALK5 pathways (Figure S6). Therefore, we sought to determine whether loss of endoglin in CDCs altered the balance of TGFβ/BMP signaling through SMAD1/5/8 (ALK1 pathway) or through SMAD2/3 (ALK5 pathway) and whether this could explain the altered pro-angiogenic properties of the CDC secretome. Endoglin-deficient and control CDCs showed no detectable difference in phosphorylation of SMAD2 or SMAD3 either in response to basal medium alone or following TGFβ stimulation (Figures 6B and 6C). However, endoglin-deficient CDCs did show significantly reduced phosphorylation of SMAD1/5/8 compared with control CDCs, which was partially rescued in response to BMP9 stimulation (Figure 6D). Therefore, the loss of pro-angiogenic effects in CM from endoglin-depleted CDCs could be a consequence of reduced BMP9 signaling or an altered balance of ALK5/ALK1 signaling. To test whether reduced BMP9 signaling was responsible for the loss of the pro-angiogenic properties of *Eng*^KO^ CM, we pre-treated CDCs (with and without endoglin depletion) with BMP9 ligand. In parallel, to determine whether the defective pro-angiogenic properties of *Eng*^KO^ CDCs were due to relative overactivity of the ALK5 signaling pathway, we treated *Eng*^KO^ CDCs with ALK5 inhibitor (SB431542). CDC-CM was harvested as before, and CM-induced angiogenic outcomes were tested using the spheroid angiogenesis assay and endothelial migration tested using the scratch-wound healing assay. Pre-treatment of CDCs with ALK5 inhibitor did not rescue the reduced pro-angiogenic or pro-migratory effects of *Eng*^KO^ CDC-CM. However, pre-treatment of CDCs with BMP9 did restore the pro-angiogenic and pro-migratory effects of *Eng*^KO^ CDC-CM to those of control CDC-CM (Figure 7). These findings show that endoglin protein responds to local levels of BMP9, and is responsible for promoting CDC-mediated pro-angiogenic paracrine effects, as summarized schematically in Figure S7.

## Discussion

The paracrine beneficial mechanisms of heterogeneous stem cell populations such as CDCs are poorly understood. Our data show that Endoglin is essential for paracrine-mediated angiogenesis by CDCs. The CDC secretome promotes an endoglin-dependent increase in endothelial tubule formation and endothelial cell migration in vitro, as well as an increased endothelial cell proliferation and higher density of mature blood vessels in vivo. Thus, the endoglin-dependent pro-angiogenic effects of CDC-CM are consistent across a wide range of in vitro and in vivo angiogenesis assays.

Endoglin is a recognized surface marker of CDCs and is also considered one of the minimal criteria for MSCs (Dominici et al., 2006). Thus, it is relevant to our findings that human MSCs from umbilical cord blood, which have been enriched for endoglin-expressing cells, lead to an increase in capillary density following delivery to a mouse MI model (Gaebel et al., 2011). We found that loss of endoglin expression in CDCs leads to reduced BMP9-dependent SMAD1/5/8 signaling responses in the CDCs, and reduced the pro-angiogenic properties of the secretome. Treatment of endoglin-deficient CDCs with BMP9 significantly increased the pSMAD1/5/8 activation response and rescued the pro-angiogenic defects of the secretome. In contrast, pre-treatment with the ALK5 inhibitor SB431542 did not rescue the angiogenic defects of *Eng*^KO^ CM, suggesting that relative overactivity of the ALK5 pathway over the ALK1 pathway following endoglin depletion did not contribute to the angiogenic defects (Figure S7). However, this is a little more complex to interpret, as ALK5 is also required for TGFβ signaling through the ALK1 pathway (Goumans et al., 2003). Nevertheless, taken together the data indicate BMP9-dependent SMAD1/5/8 signaling as a critical pathway downstream of endoglin that leads to the production of a pro-angiogenic CDC secretome.

In the heart itself we observed that increased levels of TGFβ1 and TGFβ3 are endogenously produced in the days immediately following MI, in agreement with previous data (Christia et al., 2013, Deten et al., 2001). In addition, we show that TGFβ1, BMP9, and BMP10 proteins are all present in the circulation following MI. Each of these ligands are known to interact with endoglin to promote downstream SMAD1/5/8 signaling (Castonguay et al., 2011, Lebrin et al., 2004) and can therefore interact with endoglin present on the surface of CDCs immediately following injection to the infarct border zone, thereby influencing the production of downstream paracrine factors by CDCs.

BMP9 and BMP10 have a particularly high affinity for endoglin, and BMP9 is found in an active form in the circulation (Bidart et al., 2012). Furthermore, we and others have previously shown that BMP9 signaling through ALK1 is required to maintain endoglin expression (Morikawa et al., 2011, Tual-Chalot et al., 2014). Therefore, endoglin expression on CDCs is required for normal BMP9 signaling responses, and availability of BMP9 in the media is required to maintain endoglin expression. As Endoglin is essential to generate the pro-angiogenic paracrine properties of CDCs, active BMP9 that is normally present in culture serum will be important to maintain endoglin expression during culture and expansion of CDCs (and MSCs) for clinical use. Indeed, reduced endoglin expression has been reported when MSCs are cultured in serum-free conditions (Mark et al., 2013). This is a critical factor to consider in the preparation of stem cell populations for pro-angiogenic therapy.

Recent work has shown that endoglin plays an additional role in the later stages of cardiac healing during formation of the collagenous scar tissue (Tseliou et al., 2014). Endoglin can be cleaved from the cell surface by metalloproteases such as MMP14 (Hawinkels et al., 2010). Soluble endoglin, shed from the surface of CSps, leads to reduced fibrosis when CSps are delivered in the chronic repair phase (1 month post MI) in a rat model of MI (Tseliou et al., 2014). However, Endoglin can also play a detrimental role in fibrosis. It is expressed in myofibroblasts, and reduced endoglin levels protect against adverse fibrotic responses in the aortic constriction model of hypertension (Kapur et al., 2012). Thus, in addition to its pro-angiogenic roles in the acute setting of MI, endoglin can also influence later fibrosis outcomes.

Although CDCs clearly led to a higher capillary density in the infarct border zone, we found no CDC-mediated improvement in ejection fraction or adverse left ventricular remodeling. Thus increased angiogenesis alone is insufficient to improve cardiac function in a large permanent infarct, where almost all the left ventricular free wall is affected and ultimately replaced by scar tissue. In this study great care was taken to minimize variation in infarct size, due to its major impact on outcomes (Redgrave et al., 2016). Furthermore, cardiac MRI was used to provide a robust readout of cardiac function, and investigators were blinded to treatments during the analysis. A recent systematic review of pre-clinical studies showed that the overall benefit of CDC and other cardiac stem cell treatments following MI was a small improvement in ejection fraction compared with placebo controls, but improvement was not present in all studies (Zwetsloot et al., 2016, Li et al., 2009). If improved functional outcomes are not reproducible in a standardized pre-clinical model, translation of any benefit to patients becomes even more challenging. Indeed, a recent meta-analysis of 1,871 individual patient datasets from 28 studies of acute MI patients found that intracoronary cell therapy provided no beneficial effect on left ventricular function (Gyongyosi et al., 2015). It is critical, therefore, that pre-clinical studies are reproducible to help improve the translatability of pre-clinical findings (Jones et al., 2015) and to determine the essential factors responsible for beneficial outcomes in cardiac stem cell therapy.

CDCs and MSCs are currently in clinical trials for a range of cardiac myopathies (Golpanian et al., 2016), and consistency of cell preparation/culture techniques is critical for their efficacy. Although endoglin (CD105) is used as a marker of CDCs, we now show its critical importance for maintaining their paracrine pro-angiogenic properties. Furthermore, our findings suggests that if CDCs are cultured in animal-free media for therapeutic purposes, addition of BMP9 will help to maintain endoglin expression and ensure maximal pro-angiogenic paracrine activities of these cells prior to delivery in cardiac patients.

## Experimental Procedures

### Mouse Models

All animal experiments were approved by the local ethics committee and performed in accordance with the Animals (Scientific Procedures) Act 1986 and the EU Directive 2010/63/EU. Floxed Endoglin mice and Rosa26-Cre^ERT2^ mice have been previously described (Allinson et al., 2007, Anderberg et al., 2013, Mahmoud et al., 2010). CAG-farnesyl-eGFP mice in which expression of farnesylated EGFP is ubiquitously driven by the CAG promoter were generated as part of this study (Figure S1). All mice used in this study were in a C57BL/6 genetic background.

### CDC Preparation

CDCs were cultured from *CAG-eGFP*;*Eng*^*fl*/*fl*^;*Rosa26-Cre*^*ERT2*^ mouse hearts using a previously described method (Davis et al., 2009, Messina et al., 2004). In brief, hearts from mice aged 3–6 weeks were finely minced and individual heart fragments were plated onto a fibronectin-coated dish in Iscove's modified Dulbecco's medium (IMDM) containing 20% serum and cultured for approximately 2 weeks, allowing explant-derived cells to proliferate out of the tissue fragments. Bright-phase EDCs were then harvested and plated on poly-D-lysine-coated plates with cardiosphere growth medium (65% DMEM/F12, 35% IMDM, 7% fetal bovine serum [FBS], 2% B27 [Invitrogen], 25 ng/mL cardiotrophin [Peprotech EC], 10 ng/mL epidermal growth factor [EGF; Peprotech], 20 ng/mL basic fibroblast growth factor [Promega], and 5 units of thrombin [Sigma]). After 1 week CSps were collected and cultured in cardiosphere growth medium on fibronectin-coated flasks to generate CDCs, which were used at P2. Endoglin knockout CDCs (*Eng*^KO^) were generated by the addition of 3 μM 4-OHT to the culture medium for 96 hr at P1, followed by 4 days of culture required for endogenous endoglin protein turnover with fresh media changes to allow 4-OHT washout. Cells were used immediately after endoglin protein depletion (or equivalent timings for control cells). In addition, CDCs were prepared from wild-type C57BL/6 mice and treated with 4-OHT over the same time course to determine the effect of tamoxifen alone.

### CDC Characterization and Preparation of Conditioned Medium

For characterization of surface marker expression, CDCs were incubated with antibodies against CD90 (553006, BD Biosciences), CD105 (14105185, eBioscience), CD31 (553370, BD Biosciences), CD45 (11045181, eBioscience), SCA-1 (557405, BD Biosciences), and cKIT (35117182, eBioscience). Negative staining for DAPI was used to provide a live/dead cell gate. Cells were analyzed using an LSRII flow cytometer and FACSDiva software (BD Biosciences). Positive cells were defined as the percentage of the cell population stained by more than 99.7% of isotype-matched antibody.

To prepare CM, we cultured CDCs in serum-free IMDM for 48 hr. For specific experiments, ALK5 inhibitor (SB431542, Sigma) or BMP9 (Peprotech) were added to CDCs for 24 hr and washed away with PBS prior to 48 hr of culture in fresh serum-free medium to prepare CM. Filtered CM was stored at −80°C prior to use, allowing only one freeze-thaw cycle per aliquot. Protein concentration was measured by Bradford assay (Bio-Rad).

### Endothelial Cell Culture

HUVECs (Promocell) were grown in MV endothelial growth cell medium (Promocell). Mouse lung endothelial cells (MLECs) were cultured as previously described (Anderberg et al., 2013) on 0.1% gelatin-coated flasks in MV2 endothelial growth cell medium (Promocell). HMEC-1 cells (Ades et al., 1992) were cultured on 0.1% gelatin-coated flasks in H-medium (MCDB131, 4 mM L-glutamine, 1% penicillin/streptomycin, 1 μg/mL hydrocortisone, 10% FBS) with 20 ng/mL EGF.

### 2D Matrigel Angiogenesis Assay

HMEC-1 cells were cultured on growth factor reduced (GFR) Matrigel in H-medium without EGF. Either CM or IMDM medium was added to H-medium to a final concentration of 20%. Tubule formation was imaged after 18 hr using an Axiovert200 inverted microscope fitted with an AxioCamHR digital camera. Total tubule length was quantified using NeuronJ plugin software.

### Spheroid Angiogenesis Assay

HUVECs in MV medium containing 0.25% methylcellulose were dispensed as 20-μL drops to generate spheroids that were mixed with fibrinogen (2.5 mg/mL)-aprotinin (4 U/mL, Sigma) mixture in the presence of thrombin (50 U/mL, Sigma) and spheroids cultured for 48 hr in MV medium with CM or IMDM added to a final concentration of 40%. At least 20 spheroids per condition were photographed using a Nikon ELWD 0.3/OD75 light microscope with Nikon DS-Fi1 digital camera, and length of sprouts were analyzed using ImageJ software.

### Endothelial Cell Proliferation and Viability Assays

Endothelial cell proliferation was analyzed using CyQUANTNF Cell Proliferation Assay Kit (Life Technologies) following the manufacturer's instructions. In brief, HUVECs were cultured in MV medium at 37°C for 24 or 48 hr. Subsequently, samples were analyzed using a fluorometer (Thermo Scientific) at 485-nm excitation and 530-nm emission, and data analyzed using Ascent software version 2.5. Cell viability of HUVECs cultured in CDC-CM for 24 hr and 48 hr was determined by a colorimetric 3-(4,5-dimethylthiazol-2-yl)-2,5-diphenyl tetrazolium bromide (MTT; Sigma) assay. Absorbance was measured at 550 nm by MultiSkan (Thermo LabSystems).

### Scratch-Wound Healing Assay

HUVECs were plated on a 6-well-plate at a density of 5 × 10^5^ cells and incubated in MV medium at 37°C for 24 hr. A scratch “wound” was made with a 200-μL Gilson pipette tip. Microscope images of the “wound” area were taken at 0, 24, and 48 hr and rate of “wound” closure was calculated from the mean of six assays. Images were taken using a Nikon ELWD 0.3/OD75 light microscope and a Nikon DS-Fi1 digital camera, and analyzed using ImageJ software.

### In Vivo Matrigel Plug Assay

GFR Matrigel (250 μL) was seeded with MLECs and 50 μL of CM and injected subcutaneously into flanks of adult C57BL/6 mice. Plugs were harvested after 14 days and cryosections were analyzed by immunostaining.

### Tissue Processing and Staining

Animals were humanely killed and tissue lightly fixed in paraformaldehyde (0.2%, overnight), equilibrated in 30% sucrose, and embedded in OCT compound. Cryosections were stained with anti-CD31 (553370, BD Biosciences), anti-SM22α (ab14106, Abcam), anti-αSMA (C6198, Sigma), and anti-phospho-histone H3 (06570, Millipore), and detected with secondary antibodies conjugated to Alexa 488 or Alexa 568. Sections were mounted with prolong Gold, imaged using an Axioimager M2 microscope fitted with an Apotome (Zeiss), and analyzed using ImageJ software.

### qPCR

RNA was extracted from the left ventricle using a Qiagen RNeasy Fibrous Tissue Mini Kit (Qiagen) according to the manufacturer's instructions. cDNA was prepared using a Tetro cDNA synthesis kit (Bioline) and Taq polymerase (Applied Biosystems). Commercial Taqman probes for *Eng* (Mm00468252_m1), *Bmp9* (Mm00807340_m1), *Bmp10* (Mm01183889_m1), *Tgfbeta1* (Mm01178820_m1), *Tgfbeta3* (Mm00436960_m1), *Gapdh* (Mm99999915_g1), and *Hprt1* (Mm00446968_m1) were from Life Technologies and used for qPCR on a Quantstudio 6&7 Flex Real-Time PCR system. Data were analyzed using comparative Ct (ΔΔCt) to determine relative gene expression.

### Western Blotting

CDCs were treated with BMP9 (2 ng/mL) or TGFβ1 (5 ng/mL) for 30 min before preparing whole-cell protein lysates in SDS sample buffer. HUVECs were serum starved for 4 hr before addition of CM and protein lysates prepared. Proteins were separated on 10% polyacrylamide gels and transferred onto polyvinylidene fluoride membrane before blocking with 5% powdered milk/TBST (Tris-buffered saline + Tween 20) and incubated with primary antibody to detect either phospho-SMAD1/5/8 (9516, Cell Signaling), phospho-SMAD2 (3108, Cell Signaling), phospho-AKT^Ser473^ (4060, Cell Signaling), phospho-AKT^Thr308^ (13038, Cell Signaling), AKT (4691, Cell Signaling), phospho-SMAD3 (18801, Epitomics), α-tubulin (T6199, Sigma), or β-actin (A5316, Sigma). Membranes were incubated with secondary antibody, either anti-rabbit horseradish peroxidase (HRP) or anti-mouse HRP (Dako) in blocking solution before detection using SuperSignal Chemiluminescent Substrate, and densitometric analysis using ImageQuant TL v2005 software.

### Myocardial Infarction

Acute MI was created in adult male C57BL/6 mice (12–14 weeks) as previously described (Redgrave et al., 2016, van Laake et al., 2007). Mice were pre-medicated with fentanyl/fluanisone (Hypnorm; 0.4 mL/kg) to provide intraoperative analgesia and anesthetized using isoflurane. Anesthesia was maintained using mechanical ventilation following endotracheal intubation. Left-side thoracotomy was performed through the fourth intercostal space and the left anterior descending coronary artery was ligated with a 7-0 Prolene suture. Occlusion of the vessel was verified by visible blanching of the myocardium, and only those with large infarcts progressed to the study. Mice were then subjected to two intramyocardial injections into the infarct border zone with 10 μL of PBS (MI group) or approximately 5 × 10^5^ either control CDCs or *Eng*^KO^ CDCs, with the surgeon blinded to CDC genotype. Mice in the sham group underwent left-side thoracotomy without left anterior descending artery ligation or cell injection.

### Cardiac MRI

Cardiac function was monitored after 7 and 28 days using MRI as previously described (Redgrave et al., 2016, Schneider et al., 2006). A horizontal bore 7.0T Varian system (Varian) equipped with a 12-cm microimaging gradient insert (40 gauss/cm) was used to acquire MR images. Anesthetized mice were positioned on a custom built sled (Dazai Research Instruments) with integrated electrocardiographic, respiratory, and cutaneous temperature monitoring. An SA Instruments small animal system was used for physiological monitoring and gating. A 30-mm quadrature birdcage coil (Rapid Biomedical) was used to transmit/receive the MR signal. Global cardiac function was measured from contiguous 1-mm slices covering the whole left ventricle using an ECG-triggered, respiratory gated gradient echo (FLASH) cine MR sequence (echo time 1.42 ms, repetition time 5 ms, flip angle 15°, matrix 128 × 128, field of view 25.6 mm × 25.6 mm, and 4 averages). MR images were analyzed by investigators blinded to mouse treatment using ImageJ (NIH) according to a published protocol (Schneider et al., 2006).

### RT-PCR

RNA was extracted from CDCs using a Qiagen RNeasy Micro Kit according to the manufacturer's instructions. cDNA was prepared using SuperScript III First-Strand Synthesis System (Life Technologies). Taq polymerase (Applied Biosystems) was used for RT-PCR, and PCR products were separated on an agarose gel, stained with ethidium bromide, and visualized with UV light.

### ELISA

Mouse serum was prepared by allowing 1-mL blood samples to clot for 2 hr in a serum separator tube (BD Biosciences) at room temperature before centrifugation for 15 min at 1500 × *g* and collection of supernatant. ELISAs were performed according to the manufacturer's instructions to detect TGFβ1, VEGFA (R&D); and TGFβ3, BMP9, and BMP10 (Cloud-Clone Corporation).

### Statistical Analysis

Results are presented as the mean of values ± SEM unless indicated otherwise. The number of independent experiments for each study is indicated in the figure legends. The statistical significance of differences between the mean of two groups was determined using Student's t test. When more than two experimental groups were analyzed, statistical significance of differences was first evaluated by one-way ANOVA. When multiple experimental groups were compared at different time points, data were analyzed by two-way ANOVA and post hoc t tests corrected for multiple comparisons using the Holm-Šídák method. Data were analyzed using GraphPad Prism software and a probability (p) value of less than 0.05 was considered statistically significant.

## Author Contributions

R.E.R. and S.T.-C. designed and conducted most of the experiments. B.J.D., D.H., E.S., M.M.A., and D.G. performed experiments. H.M.A. and A.M. conceived and coordinated the project. H.M.A., R.E.R., and S.T.-C. prepared the manuscript.

## Supplemental Information

Document S1. Figures S1–S7

Document S2. Article plus Supplemental Information
