# Supplementary material for: Cardiosphere-Derived Cells Require Endoglin for Paracrine-Mediated Angiogenesis
Source: Stem Cell Reports. 2017 May 9;8(5):1287–98. doi: 10.1016/j.stemcr.2017.04.015 (PMC5425789; doi:10.1016/j.stemcr.2017.04.015)
Supplement: Document S1. Figures S1–S7 [file mmc1.pdf]

Stem Cell Reports, Volume *8*

Supplemental Information

Cardiosphere-Derived Cells Require Endoglin for Paracrine-Mediated Angiogenesis

Rachael E. Redgrave, Simon Tual-Chalot, Benjamin J. Davison, Esha Singh, Darroch Hall, Muhammad M. Amirrasouli, Derek Gilchrist, Alexander Medvinsky, and Helen M. Arthur

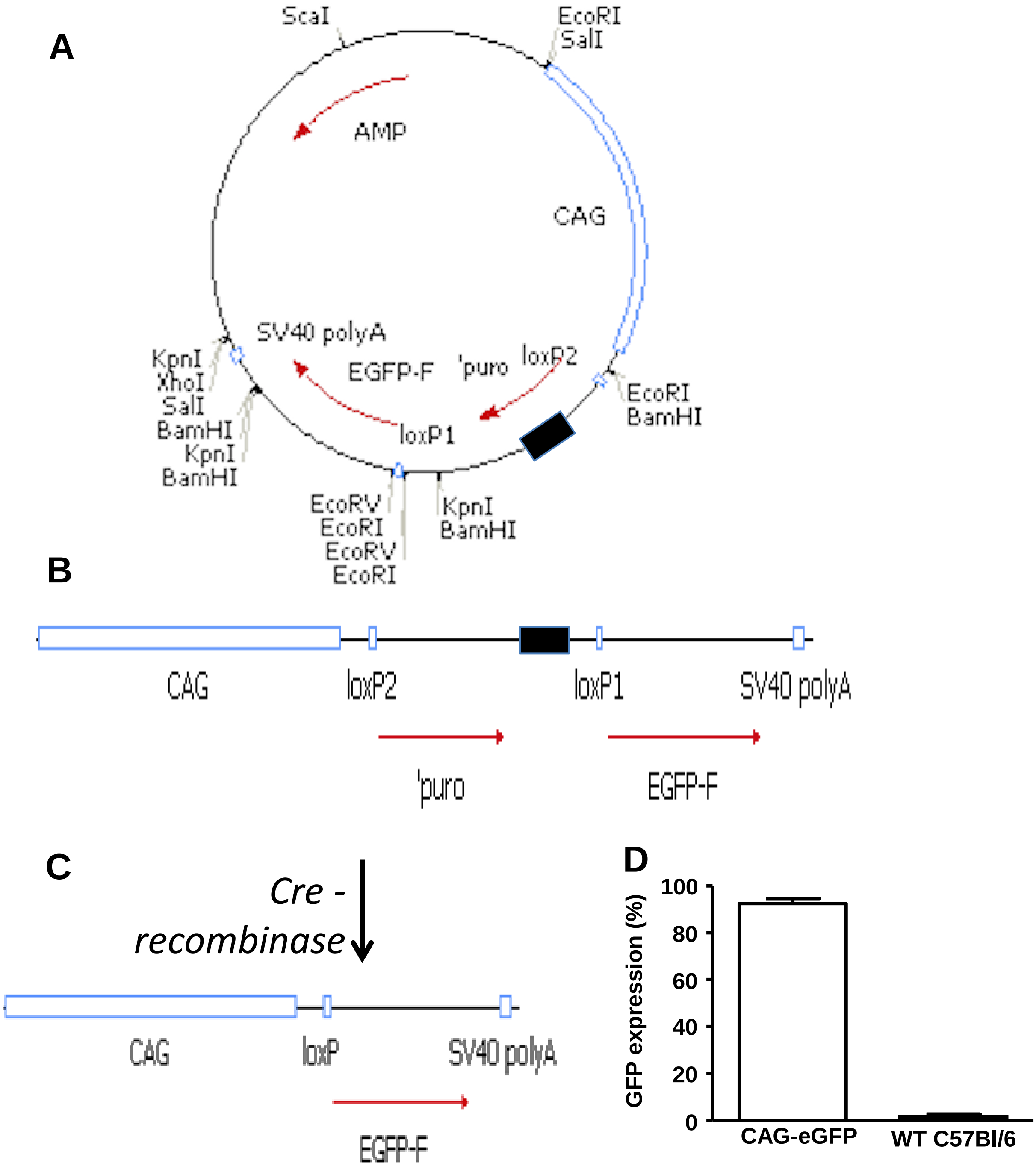

Figure S1 Generation of *CAG-farneslylated eGFP* transgenic mouse line.
A-C: The *CAG-farnesylated-eGFP* transgenic mouse line was generated using the plasmid illustrated in which the CAG promoter (open blue box) was cloned upstream of a puromycin acetyl transferase (Puro) STOP cassette (closed black box) and the coding region for farnesylated eGFP (Clontech) was cloned downstream (A). The Puro-STOP cassette was floxed by 2 wild-type loxP sites and was excised by Cre/loxP recombination in vivo by crossing with female ZP3-Cre transgenic mice, resulting in constitutive expression of farnesylated eGFP (B,C). Constitutive eGFP expression in over 92% of CDCs prepared from this line was confirmed by FACS analysis (D).

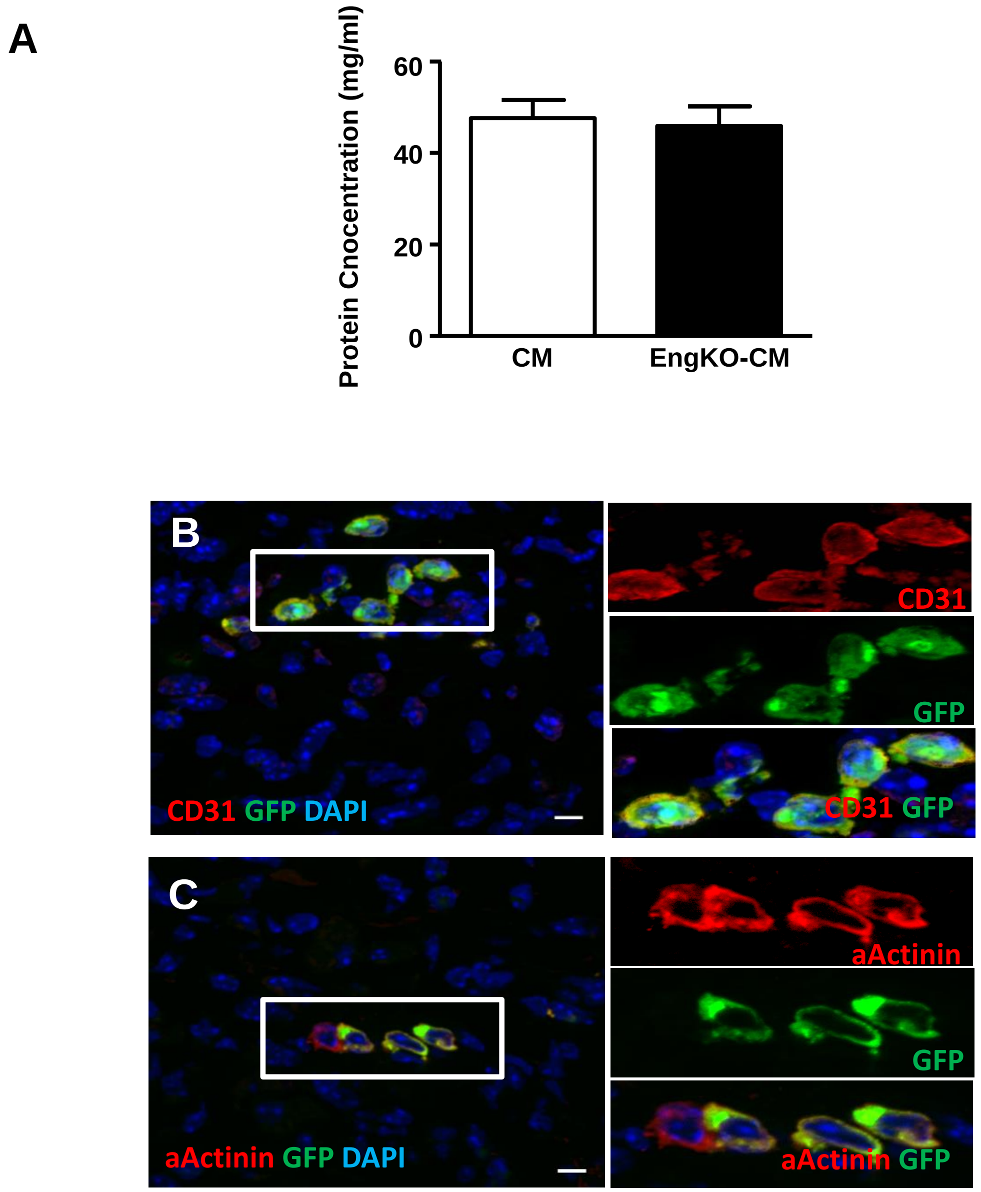

Figure S2 CDC properties of conditioned media and cell differentiation potential in vivo

A Protein concentration in conditioned media prepared from CDCs is equivalent in CM and *Eng*^{KO} CM. Protein concentration for each sample of CDC conditioned media (n=14) was quantified by Bradford assay.

B-C Tracking GFP labelled CDCs in recipient hearts following myocardial infarction**.** Rare GFP-positive CDCs from the *CAG-farnesylated-eGFP* transgenic mouse donor line were observed 4 weeks after injection. Genetically tagged GFP positive CDCs that had differentiated to express the endothelial marker CD31 (B) or the cardiomyocyte marker alpha-actinin. Scale bar=10um

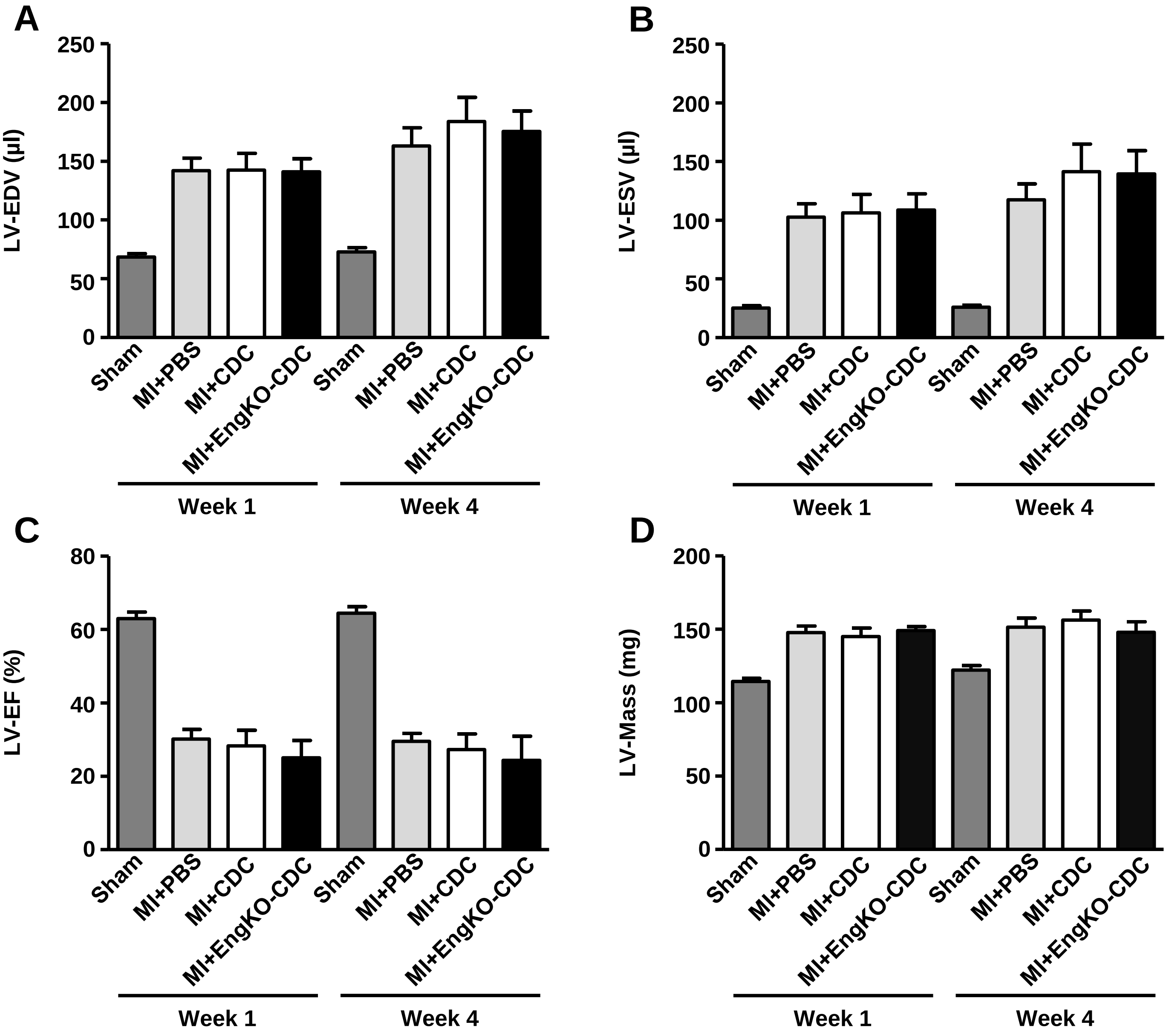

Figure S3 Cardiac function following myocardial infarction with and without delivery of control and *Eng*^{KO} CDCs to the infarct border zone

A,B: Left ventricular end diastolic volume (LV-EDV) and end systolic volume (LV-ESV) increases at 1 and 4 weeks following MI in adult male C57BL/6 mice. Outcomes were similar in all MI groups irrespective of whether PBS, control CDCs or *Eng*^{KO} CDCs were delivered to the infarct border zone.

C,D: Left ventricular ejection fraction (LV-EF) is reduced whilst myocardial mass (LV-mass) increases at 1 and 4 weeks following MI in adult male C57BL/6 mice. These changes were similar in all MI groups irrespective of whether PBS, control CDCs or *Eng*^{KO} CDCs were delivered to the infarct border zone.

All data are plotted as means +/- SEM. Group sizes: Sham, N= 11; MI +PBS, N= 18; MI+CDCs, N= 11; MI+*Eng*^{KO}-CDCs, N=8.

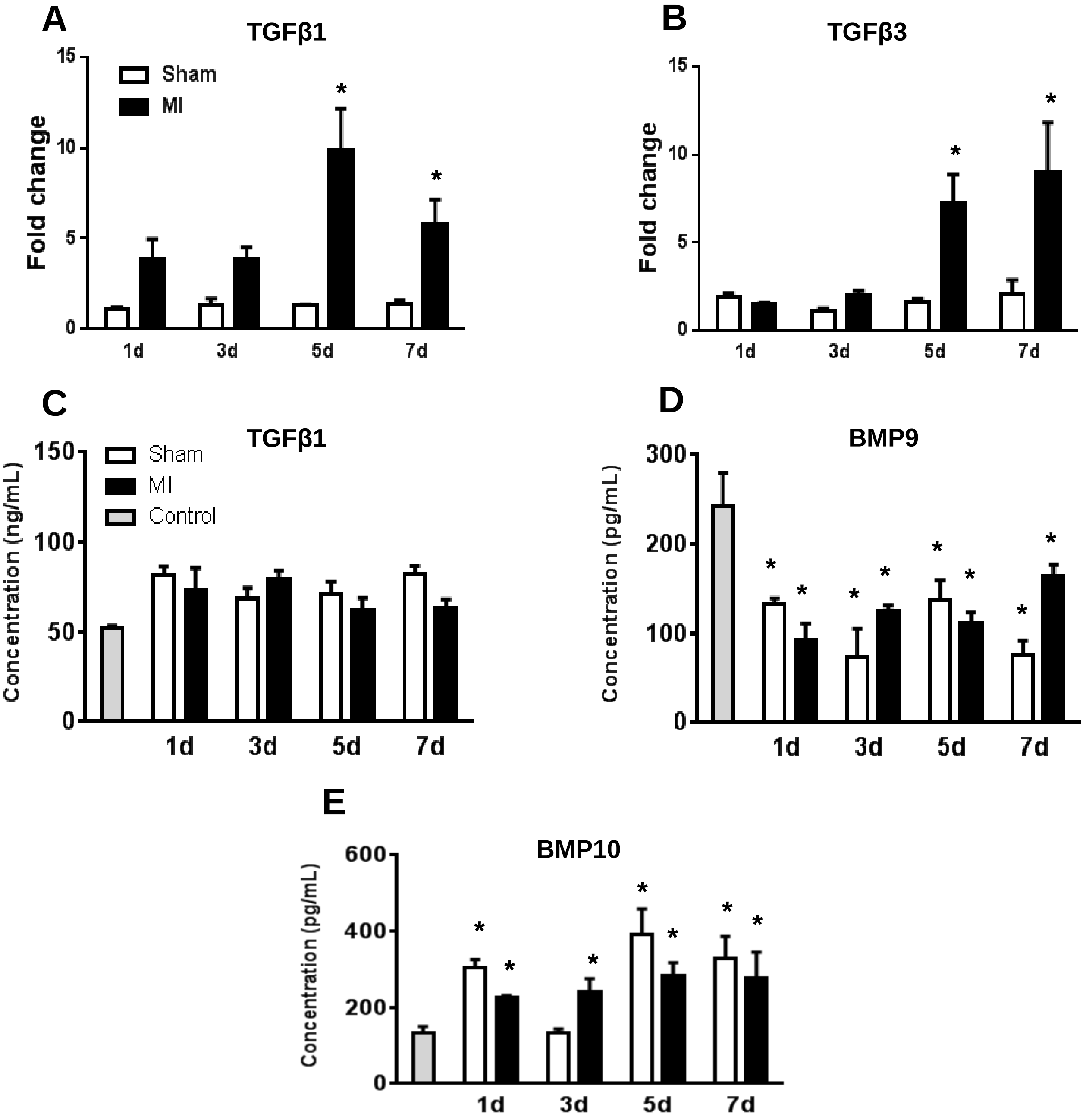

Figure S4 Dynamic changes in expression of TGFβ family ligands following myocardial infarction.

A,B: Analysis by qPCR shows dynamic changes in expression of TGFβ1 and TGFβ3 transcripts in left ventricular free wall following myocardial infarction (MI). Transcript levels in MI and sham hearts are calculated relative to normal hearts from naive age-matched male C57BL/6 mice. TGFβ1 expression peaks in the infarcted heart tissue at day 5 following MI, whilst TGFβ3 continues to increase at day 7. Data analysis by two-way ANOVA show that MI caused a significant increase in TGFβ1 and TGFβ3 expression. Post hoc t tests were corrected for multiple comparisons; *$p<0.05$ (n=6/group).
C-E: ELISA of mouse serum shows concentration of circulating TGFβ1, BMP9 and BMP10 protein following myocardial infarction or sham surgery. Circulating protein levels are calculated relative to those from naive age-matched male C57BL/6 mice. Data was analysed by two-way ANOVA and circulating levels of BMP9 showed significant decrease whilst BMP10 showed significant increase following surgery. Post hoc t tests were corrected for multiple comparisons; *$p<0.05$ (n=4/group).

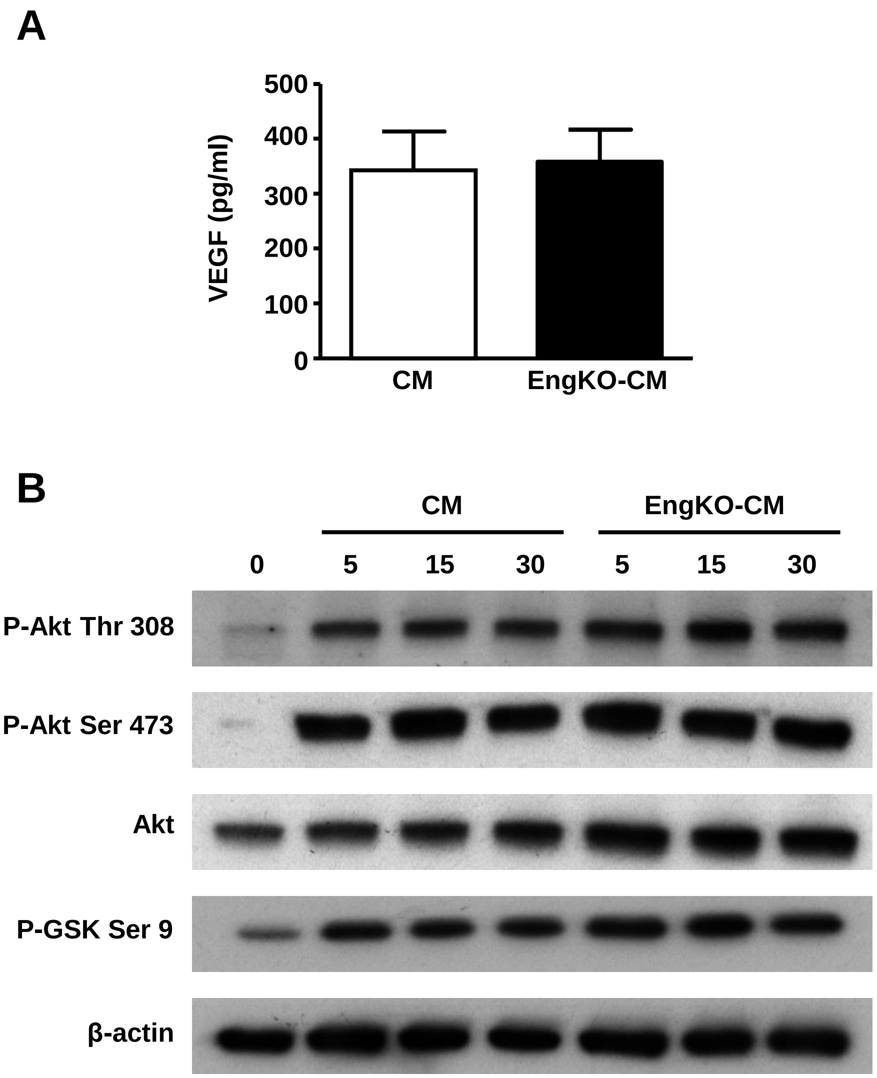

Figure S5 Loss of endoglin in CDCs has no effect on VEGF levels in conditioned media (CM) or on stimulation of AKT pathway in CM-treated endothelial cells .

A: Concentration of VEGF in CDC-CM is similar in control-CM and EngKO-CM, plotted as mean +/-SEM; N=15/group.

B: Treatment of HUVECs with control CM and Eng^{KO} CM for 5, 15 and 30 minutes led to similar activation of proteins downstream of VEGF signalling. Representative western blot showing phospho-AKT serine473, phospho-AKT threonine 308 and phospho-GSK levels are present at comparable levels following stimulation of HUVECs with control CM and Eng^{KO} CM, consistent with their similar VEGF content.

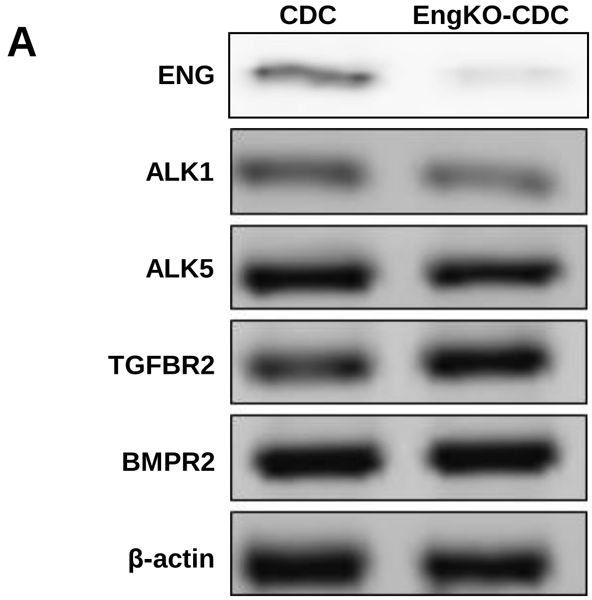

B Primers used for RT-PCR.

ENG	F: CAATGCCAGCATTGTCACCTCC<br>R: AGAGGCTGTCCATGTCGATGCA
ALK1	F: GACCTTGGGGAGCTTCAGA<br>R: TGCAGAAGGATCTATAGCAGCA
ALK5	F: GCACCATCTTCAAAAACAGGGG<br>R: GCCAAACTTCTCCAAACCGACC
TGFBR2	F: GGAAGTCTGCGTGGCCGTGTGG<br>R: CTATGGCAATCCCCAGCGGAGG
BMPR2	F: CTCAGAATCAAGAACGGCTGTG<br>R: TGAATGAGGTGGACTGAGTGGT
β-Actin	F: TGAACCCTAAGGCCAACCGTG<br>R: GCTCATAGCTCTTCTCCAGGG

Figure S6 Receptor components of the TGFβ and BMP9 signalling pathways are present in CDCs. TGFBR2, BMPR2, ALK1 and ALK5 receptors for BMP/TGFβ signalling are all expressed in control and Eng^{KO} CDCs as assessed by rtPCR (A), using primers shown (B).

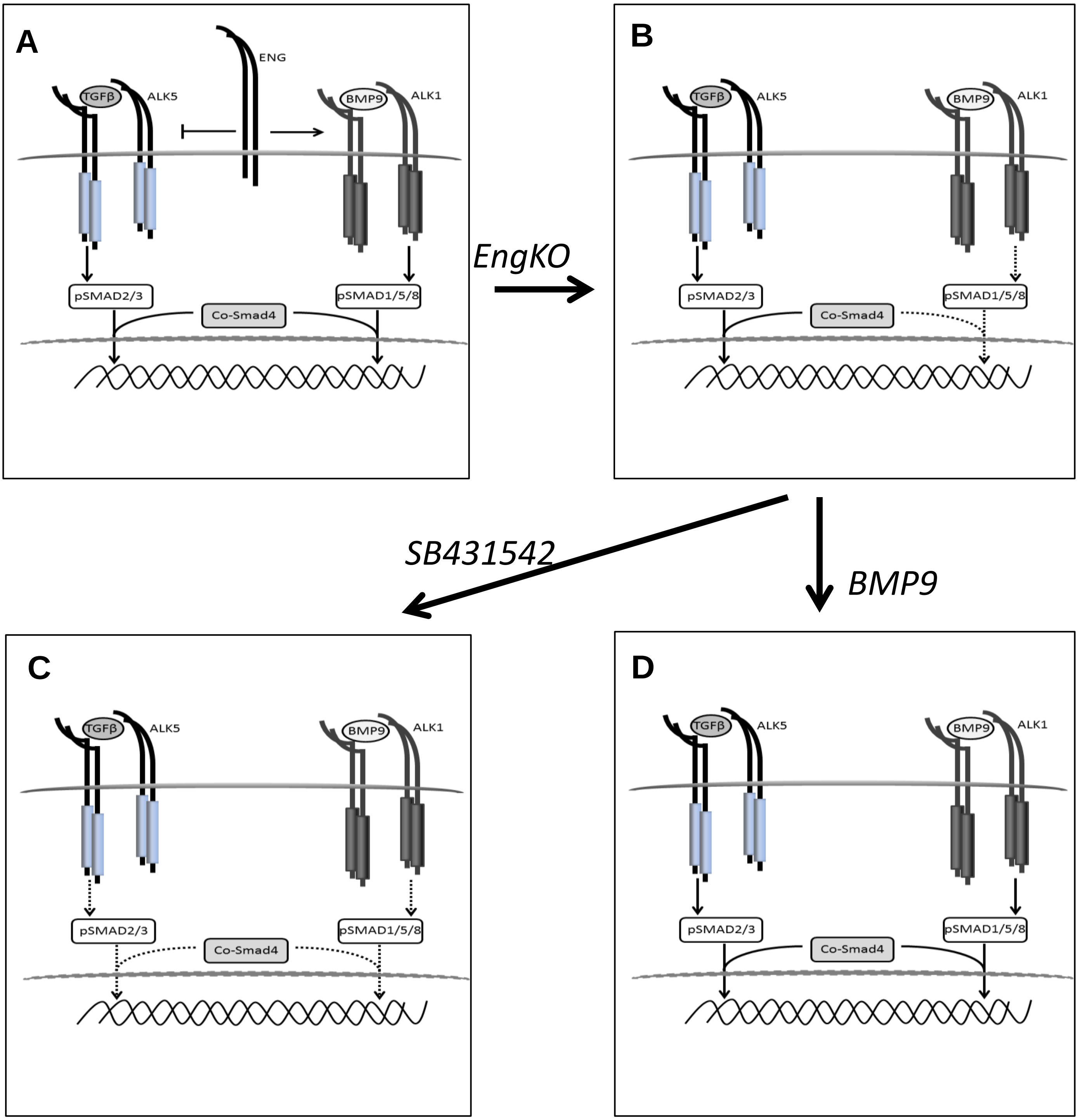

Figure S7 Summary of signalling defects in CDCs in the absence of endoglin, and rescue by BMP9.

A: Wild type CDCs show activation of SMAD2/3 and SMAD1/5/8 pathways in response to exogenous ligands. Activation of these pathways is stimulated by TGFβ (present in the heart and circulation) and BMP9 (present in the circulation).

B: Loss of endoglin leads to reduced activation of SMAD1/5/8 pathway but has no detectable effect on the SMAD2/3 pathway.

C: ALK5 Inhibitor (SB431542) decreases activation of the pSMAD2/3 pathway but does not restore the SMAD1/5/8 pathway.

D: BMP9 treatment rescues activation of the SMAD1/5/8 pathway, which in turn rescues the pro-angiogenic defects of Eng^{KO}-CM.
